# X-Linked Agammaglobulinemia Leading to Chronic Obstructive Lung Disease

**DOI:** 10.7759/cureus.32470

**Published:** 2022-12-13

**Authors:** Paris Bean, Pushan Jani

**Affiliations:** 1 Pulmonary Medicine, McGovern Medical School, Houston, USA

**Keywords:** chronic lung disease, lung pathology, lung, copd, agammaglobulinemia

## Abstract

X-linked agammaglobulinemia (XLA) is a rare primary immunodeficiency disorder. It occurs in around one in 200,000 live births and is caused by mutations in the Bruton Tyrosine Kinase (BTK) gene leading to B lymphocyte deficiency and increased susceptibility to infection. Infection is the most common initial clinical presentation, followed by family history and neutropenia. Even in patients with a positive family history, only 34% of patients were diagnosed before clinical symptoms arose. Over half of patients are diagnosed by two years of age. Treatment is aimed at replacing immunoglobulin using intravenous immunoglobulin (IVIG) or subcutaneous immunoglobulin (SCIG) and prophylactic antibiotics to prevent infections. Despite these therapies, patients still suffer from repetitive infections. Another significant source of morbidity in patients with XLA is a chronic lung disease. By the time of diagnosis, 62% of patients had at least one case of pneumonia. We describe the case of a patient who has developed an accelerated course of chronic obstructive pulmonary disease (COPD) secondary to pre-existing X-linked agammaglobulinemia and recurrent respiratory infections.

## Introduction

Primary immunodeficiencies are rare inherited defects of the immune system that lead to increased susceptibility to infections. Of these, X-linked agammaglobulinemia (XLA) was the first to be identified to be connected to a genetic cause [1]. Most children become symptomatic by 6-12 months of age when the mother’s immunoglobulins clear the child’s system. Diagnosis is made usually after children become symptomatic, and 50% of patients have had serious infections by the time of diagnosis. Recurrent bacterial upper and lower respiratory tract infections are the most common manifestation [2]. These infections are typically caused by encapsulated pyogenic bacteria such as *Streptococcus Pneumoniae*, *Haemophilus* *Influenzae* type B, *Streptococcus pyogenes*, and *Pseudomonas* species. Treatment is aimed at replacing immunoglobulins either with IVIG (intravenous immunoglobulin) or subcutaneous immunoglobulin (SCIG) [3]. 

In patients who have primary immunodeficiency disorders, including XLA, lung infections and chronic lung diseases such as bronchiectasis secondary to recurrent infections are the most commonly reported causes of death [4]. While prompt treatment with immunoglobulin has been shown to reduce infection, it does not reduce the risk of developing chronic lung diseases like bronchiectasis [5]. Intensive secretion clearance therapy and regular pulmonary function monitoring could help reduce the risk of pulmonary insufficiency in these patients [6,7]. We report the case of a patient who continues to have recurrent lung infections despite having regular treatment with IVIG and SCIG. Recurrent infections have led to chronic lung disease in the form of severe obstruction and significant air trapping leading to chronic respiratory failure with hypoxemia requiring dependence on home oxygen use. This case report demonstrates the urgent need for developing alternative therapies to prevent severely morbid chronic lung diseases. 

## Case presentation

A 34-year-old man presented with one month of progressively worsening shortness of breath associated with cough and green sputum production. He has a past medical history of X-linked agammaglobulinemia (XLA). He was first diagnosed at two-and-a-half years of age after repetitive infections. Three boys in his maternal family had died in early childhood (before the age of three) secondary to infections without being diagnosed. His maternal cousin had also received a diagnosis of XLA shortly after the patient. The patient started treatment with IVIG at the time of diagnosis and eventually was transitioned to Hizentra (human immune globulin) subcutaneously. He remained healthy until his early 20s when he started smoking and had concurrent repetitive bouts of bacterial pneumonia. He continued to smoke one pack of cigarettes per day for five years before quitting. During this time, he did not require supplemental oxygen therapy in between episodes of pneumonia. He continued to have good exercise tolerance. He also continued using subcutaneously injected human immunoglobulin. He had received all recommended childhood vaccinations, including recent vaccination against COVID-19. He also received annual influenza vaccination. 

His condition worsened at the age of 34 when he had bacterial pneumonia and was eventually diagnosed with bronchiectasis based on computed tomography (CT) scans of the chest and lung function testing. He presented to the hospital with increasing shortness of breath both at rest and with activity and a cough productive of green sputum. He was treated with intravenous levofloxacin, intravenous methylprednisolone, and nebulized ipratropium and albuterol. He was eventually discharged on Prednisone, Nebulized Albuterol, and Umeclidinium/Vilanterol inhaler. He also was discharged on home oxygen at 2 liters per minute via nasal cannula. 

**Figure 1:**
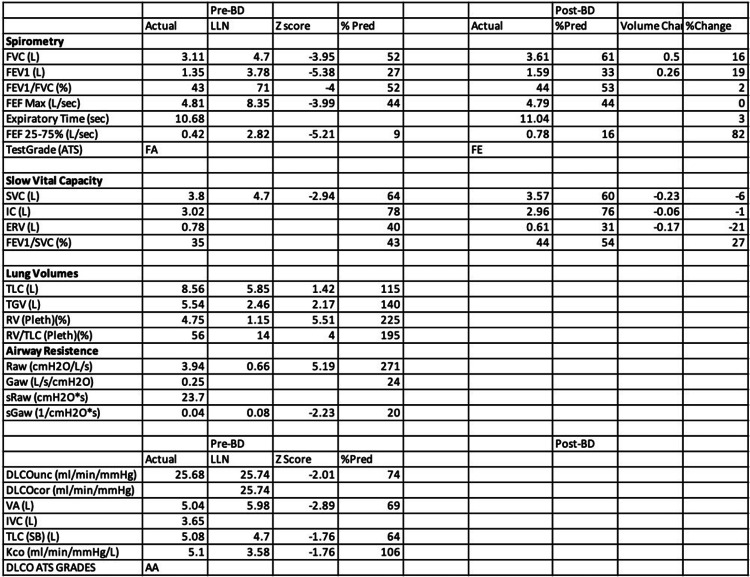
Pulmonary function testing FVC: Forced vital capacity; FEV1: Forced expiratory volume; FEF: Forced expiratory flow; SVC; Slow vital capacity; IC: Inspiratory capacity; ERV: Expiratory reserve volume; TLC: Total lung capacity; TGV: Thoracic gas volume; RV: Residual volume; DLCO: Diffusing capacity for carbon monoxide; VA: Alveolar volume; Kco: Carbon monoxide transfer coefficient

**Figure 2:**
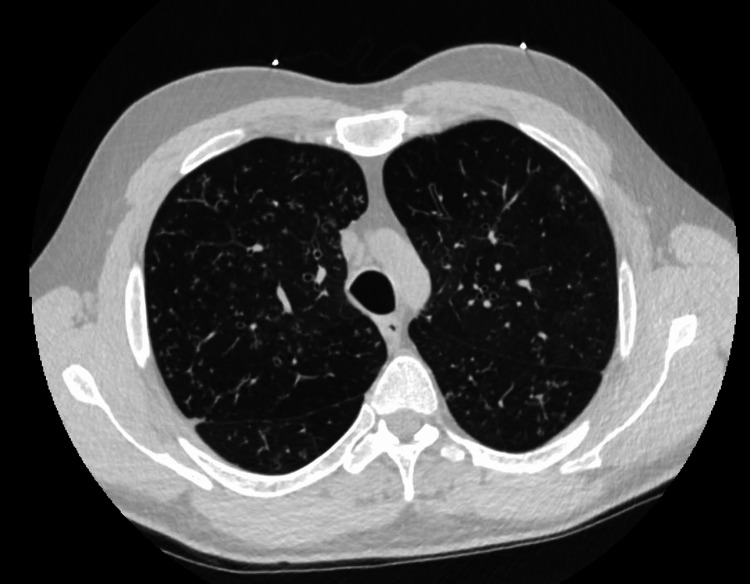
CT scan of the chest showing air trapping and tree-in-bud opacities Tree in-bud opacities and bronchiectasis of the airways

Pulmonary function testing was performed and showed very severe airway obstruction (FEV1/FVC ratio: 43, FEV1: 1.35 liters (27% predicted), with air trapping (RV: 4.75 liters, 225% predicted) and a mild diffusion defect (Corrected DLco: 25.68 ml/min/mmHg (74% predicted). Full PFT results are shown in Figure 1. Patient was continued on home oxygen and bronchodilators. However, he continues to have recurrent hospital admissions for episodes of worsening shortness of breath and productive cough. He was treated with empiric antibiotics, which did not improve his condition. CT Computed Tomography (CT) scan of the chest (Figure 2) showed diffuse tree-in-bud opacities and lower lobe bronchiectasis. He underwent flexible bronchoscopy with bronchoalveolar lavage, which grew Cladosporium, and transbronchial lung biopsies showed benign lung parenchyma with focal peribronchial chronic inflammation. Despite being on 10 mg of oral prednisone daily, along with the inhaler therapy as mentioned above, he continues to have significant dyspnea on minimal exertion and is now being evaluated for possible lung transplantation. His decreased lung function continues to be a large physical and emotional burden. 

## Discussion

In patients with X-Linked agammaglobulinemia, severe bronchiectasis and cor pulmonale are the most common causes of morbidity and mortality. A study looking at a cohort of XLA patients showed that 13.1% of patients had chronic lung disease present at the time of diagnosis, and around 51% of patients developed chronic lung disease after 40 years of follow-up. Patients with XLA have a cumulative risk of 47% developing chronic lung disease [5]. The development of chronic lung disease has been demonstrated in other immunodeficiencies, such as human immunodeficiency virus (HIV) infection [8].

XLA patients often develop bronchiectasis and other chronic lung diseases secondary to repeat infections leading to airway inflammation and structural damage [9]. The most common pathogens isolated in these individuals are *Haemophilus influenzae* and *Streptococcus pneumoniae*, followed by *Pseudomonas* species, *Staphylococcus* species, *Klebsiella pneumoniae*, *Branhamella catarrhalis*, and *Pneumocysis jiroveci* [5]. As a result of these repeat infections, both local and systemic inflammation increases, and this leads to ongoing damage from phagocytes, neutrophils, and eosinophils [10]. This ongoing damage is one proposed mechanism behind the pathogenesis of chronic lung disease in patients with XLA.

The use of IVIG and SCIG for patients with XLA has been shown to decrease the number of infections significantly. However, even with the significant decrease in infections, there was no change in the development of chronic lung disease [5], as demonstrated in this case report. Despite being on immune replacement therapy since he was around three years old, our patient has still had numerous pneumonia episodes and is now suffering from bronchiectasis and severe airway obstruction.

It is hypothesized that the lack of mucosal IgA (immunoglobulin A) cannot be compensated for by IgG (immunoglobulin G) replacement treatment, and thus patients still have repeat pulmonary infections even after treatment [5]. One study found that lower IgA serum levels were significantly correlated with lung disease in a patient with primary immunodeficiency, including XLA. This study also found that these patients had higher bacterial colonization associated with lower serum levels of IgA [11]. More research in the etiology and pathogenesis of chronic lung disease in patients with XLA and other primary immunodeficiencies is needed to reduce the burden of disease in these patients. Respiratory infections and chronic lung disease remain the leading cause of death in these patients [4]. Patients have been treated with lung transplantation for severe end-stage lung disease secondary to recurrent infections from X-linked agammaglobulinemia [12].

## Conclusions

Chronic lung disease is a significant cause of morbidity and mortality in patients with X-linked agammaglobulinemia (XLA), even patients that are on long-term immunologic therapy. There remains a high need for research in this area to decrease the rate of chronic lung disease and mortality in patients with XLA. Patients with end-stage lung disease with oxygen dependence secondary to X-linked agammaglobulinemia (XLA) should be referred early for evaluation of lung transplantation, as double lung transplantation has been successful in these patients.
